# Prevalence of Geohelminthic Infection and Its Risk Factors Among School Children in Thiruvananthapuram, Kerala, India

**DOI:** 10.7759/cureus.34116

**Published:** 2023-01-23

**Authors:** Puhalenthi K, Chandrababu C, Maniprabhu S, Vijayakumar K, Thomas Mathew, Mini SS

**Affiliations:** 1 Community Medicine, K.A.P. Viswanatham Government Medical College, Trichy, IND; 2 Paediatrics, Trichy SRM Medical College Hospital and Research Centre, Trichy, IND; 3 Community Medicine, Government Medical College, Thiruvananthapuram, Thiruvananthapuram, IND

**Keywords:** risk factor, school children, soil-transmitted helminths, kerala, geohelminth

## Abstract

Introduction

Geohelminthic infections are the most common infections worldwide and affect the poorest and most deprived communities. School-age children typically have the highest intensity of worm infection. Currently, information on the prevalence of geohelminthic infestations among schoolchildren is scarce in Kerala. As a result, it would be worthwhile to investigate the prevalence and risk factors of a geohelminthic infestation among schoolchildren in Kerala's Thiruvananthapuram district.

Materials and methods

A community-based cross-sectional study was conducted among 454 primary school children (5-10 years) in the Thiruvananthapuram district. Samples were selected by stratified multi-stage sampling, and the data were collected using a pre-tested, semi-structured questionnaire. The stool examination was performed for each study subject, and the data were recorded.

Results

The overall prevalence of geohelminthic infections was 9.69% (95% confidence interval: 5.62-14.61), with Ascaris (A.) lumbricoides accounting for 5.7%, Trichuris (T.) trichiura accounting for 3.3%, and hookworm accounting for 0.7%. Children residing in rural areas (odds ratio (OR): 40.16; 95% confidence interval (CI): 5.28-305.3), studying in government schools (OR: 3.06 with 95% CI: 1.55-6.05), and using piped water as a source of drinking water (OR: 1.96; 95% CI: 1.01-3.77) were found to be significant and had emerged as risk factors for geohelminthic infestation. Children wearing footwear while playing outside (OR: 0.77; 95% CI: 0.39-1.5) and having personal hygiene class in school (OR: 0.34; 95% CI: 0.14-0.83) were found to be significant and had emerged as protective factors for geohelminthic infestations.

Conclusion

The prevalence of geohelminthic infestations among schoolchildren in this study was 9.7%. The individual species prevalence of A. lumbricoides, T. trichiura, and hookworm were 5.7%, 3.3%, and 0.7%, respectively. In addition to geohelminthic infestation, this study discovered a prevalence of 0.9% with Enterobius vermicularis. The factors found to be significantly associated with geohelminthic infestation after doing multivariate analysis were children belonging to government schools, residing in rural areas, and using piped water as a source of drinking water.

## Introduction

Geohelminths, also known as soil-transmitted helminths (STHs), are a family of intestinal parasites that infect people when they come into touch with their warm, damp, or warm-blooded eggs or larvae. This is a member of the Nematoda class, which also contains whipworms (Trichuris (t.) trichiura), roundworms (Ascaris (A.) lumbricoides), and two hookworms (Ancylostoma duodenale and Necator americanus). As long as there remains poverty in the developing world, people will continue to be at risk of having soil-transmitted helminth (STH) infections [1]. One of the most significant neglected tropical disease clusters of our day is STH infections on a global scale [2].

Worldwide, STH infections affect more than 1.5 billion people or 24% of the world's population [3]. Children who live in regions where these parasites are heavily transmitted - more than 270 million preschoolers and more than 600 million school-age children - need treatment and prevention measures [3]. Around 819 million individuals worldwide have Ascaris lumbricoides infections, 464.6 million have Trichuris trichiura infections, and 438.9 million have hookworm infections, per the Global Burden of Disease (GBD) research from 2010. 70% of these infections occur in Asia while India accounts for a higher percentage of overall individual infections with one or more STH than any other country, at roughly 21% [4]. With variable prevalence rates for specific parasites in India, the reported prevalence of STH ranges from 12.5% to 66% [5]. According to GBD 2010, around 71 million (12%) Indians have hookworm infections, 73 million (12%) have T. trichiura, and 140 million (17%) have ascariasis [6]. According to research conducted in the Thiruvananthapuram district of Kerala in 2002, the prevalence was 22.27%, with A. lumbricoides accounting for 74.41% of cases, hookworm accounting for 9.7%, and other forms accounting for 6.97% [7].

The burden of STH infections is commonly calculated using disability-adjusted life years (DALY), which includes both years of life lost through premature death (YLL) and years of life lived with disability (YLD). Around 4.98 million YLDs were attributed to STH globally in 2010, with 65% of those being attributed to hookworm, 22% to A. lumbricoides, and the remaining 13% to T. trichiura. Asia is where the great majority of these occur [4].

The most severe infections in children between the ages of 5 and 15 are caused by A. lumbricoides and T. trichiura, whose severity and frequency decrease as they age. Although severe hookworm infections can sometimes occur in children, they frequently persist in adults and can even affect elderly individuals. The risk of contracting STH infection and higher prevalence cannot be attributed to a single factor alone; rather, they are the result of the coexistence and amalgamation of numerous biological, social, behavioral, and environmental factors, such as low socioeconomic status, a lack of clean drinking water, unsanitary hygiene practices, inadequate fecal disposal methods, and a lack of hygienic behavior at both the individual and community levels. The higher risk of STH transmission is also attributed to the wide distribution of parasites within human societies and the ineffectiveness of health services.

Of all age groups, school-aged children often suffer the worst worm infections. Malnutrition, iron deficiency anemia, malabsorption syndrome, intestinal obstruction, chronic diarrhea, rectal prolapse, respiratory issues, and inadequate weight gain are among the morbidities brought on by STH [8]. Children's health, nutrition, cognitive development, learning, and access to and success in education are all adversely impacted by chronic STH infection. Kerala has seen an epidemiological transition from communicable diseases to non-communicable diseases before many other Indian states. In the state, non-communicable diseases are a serious problem. STH remains widely ignored by the medical and state-level communities in spite of the relevance it has for education, the economy, and public health.

To comprehend the significance of STH infection in a community and to design control measures, epidemiological statistics are crucial. This study's goal is to determine the prevalence of geohelminth infestation in school children (5-10 years) in the Thiruvananthapuram district of Kerala state in India.

## Materials and methods

Study design

This was a community-based cross-sectional, analytical study.

Study place and population

The current study was conducted among children in the age group of 5-10 years who attended primary schools located in Thiruvananthapuram district, Kerala state, in south India.

Sampling technique and sample size

This study was done with a multi-stage sampling technique. At first, all primary schools (Standards 1-5) located in the geographical area under the jurisdiction of the district are categorized into government schools, government-aided schools, and government-unaided (private) schools. A total of 12 schools were randomly selected and included in this study.

The sample size was based on a predicted prevalence of STH of 34.56% [9] among schoolchildren with a 5% absolute error and a 95% confidence level. Accounting for a 20% non-response rate and a design effect of 2, the final estimated minimum sample size was 437. The current study took 454 samples.

The total sample size was divided proportionately into government, aided, and unaided sectors (categories) as per the Government of Kerala school statistics report. The total percentage of students was 48.3%, 41.9%, and 10.48% from government, aided, and private schools, respectively.

In the second stage, a stratified sampling technique with a probability proportional to size (PPS) sampling method was used, taking into account the factors of school resident, school type, student's school grade, and class section. From the selected schools (Government/Aided/Private), from each class, one division (example: I-A) from each standard (1st to 5th standard) was identified at random, and all the children in the selected division were included in the study.

In the third stage, 218 children from government, 184 children from government-aided schools, and 52 children from private schools were chosen at random from a selected class section of the student's school grade (using a random number table).

Eligibility criteria

A randomly selected parent or guardian of children in selected sections of the 1st to 5th grades was invited to an informational session where the objectives of the study were fully explained, including the risks and benefits of participating in the study. Those who provided willing consent were enrolled in the study; otherwise, they were not.

Data collection and data tool

The data were collected by visiting the selected schools, and stratification of the standards from 1st to 5th was done. Data on sociodemographic characteristics and variables related to hand-washing, footwear-wearing habits, and the frequency of meat intake of the children were collected using a pretested, semi-structured questionnaire administered by face-to-face interview.

Once the interview was completed, a "stool collection kit" was given to the parents with prior instructions on the sterile stool collection technique. The kit was properly labeled with the child’s numeric code and included a pair of disposable paper gloves for collecting the stool specimen, one wooden spatula for scooping the stool specimen into the container, and one non-sterile, wide-mouthed, leak-proof container with a cover in a plastic bag for bringing the stool specimen to the investigators. On the next day, the stool samples were collected from the students and transferred to the microbiology laboratory at the Government Medical College in Thiruvananthapuram.

The presence of Nematode ova was determined in saline and iodine direct wet mount preparations. Concentration methods can be based on the sedimentation principle; formaldehyde is used for concentrating cysts, eggs, and larvae.

Ethical clearance

Ethical clearance was obtained from the institutional ethical committee of the Government Medical College in Thiruvananthapuram, Kerala, India (IEC No. 08/27/2012/MCT). Before obtaining written informed consent, the purpose of the study, and the entirely voluntary nature of participation were clearly explained to all students and their parents. Stool examination results were revealed to all participants, and appropriate drug treatment was given to students infected with STH.

Statistical analysis

The collected data were entered into and analyzed using the Statistical Package for Social Sciences (SPSS; IBM Corp., Armonk, NY). Frequencies, percentages, and measures of central tendency were calculated. A bivariate analysis of independent variables (Pearson’s chi-square and Fisher’s exact tests) with STH infection was done to select a subset of predictor variables. A multivariate logistic regression model was done to observe and identify determinant factors associated with STH. All variables with a P-value less than 0.2 were included in the final model. A P-value of less than 0.05 was considered statistically significant.

## Results

A total of 490 parents of students were approached. Thirty-six parents of students did not give consent for the study. The non-respondent rate was 8.0%. The remaining 454 children were included for data collection and analysis.

Table 1 describes the socio-demographic variables of the study participants. The mean age (SD) of the children was 8.1 (1.59 years). About 51.5% (n = 234) of the study subjects were female. Forty-seven point six percent (47.6%; 216) of the students were below the poverty line (BPL). Rural areas were home to 63.4% (288) of the students.

**Table 1:** Sociodemographic variables and association with geohelminthic infestations (n = 454) STH: Soil-Transmitted Helminthiasis, OR: Odds Ratio, CI: Confidence Interval Test used: Pearson chi-square, df = 1, p-value significance level <0.05 Odds Ratio (OR) > 1 denotes the factor is a risk and OR < 1 denotes the factor is protective.

S. No	Variables	Category	STH subjects (n= 48)	Non-STH subjects (n= 406)	p-value	Crude OR (95% CI)
1	Gender	Male (n=220)	23 (10.5%)	197 (89.5%)	0.937	0.976 (0.536-1.776)
		Female (n=234)	25 (10.7%)	209 (89.3%)		
2	Age category	≤ 8.5 years (n=232)	27 (11.6%)	205 (88.4%)	0.45	0.793 (0.434 – 1.449)
		>8.5 years (n=222)	21 (9.5%)	201 (90.5)		
3	Religion	Hindu (n=264)	29 (10.9%)	235 (89.1%)	0.142	---
		Muslim (n=58)	2 (3.44%)	56 (96.5%)		
		Christian (n=132)	17 (12.9%)	115 (87.1%)		
4	School type	Government (n=218)	31 (14.2%)	187 (85.8%)	0.015	2.136 (1.146-3.981)
		Aided and private (n=236)	17 (7.2%)	219 (92.8%)		
5	Residence	Rural (n=288)	47 (16.3%)	241 (83.7%)	0.000	32.17 (4.3- 235.5)
		Urban (n=166)	1 (0.6%)	165 (99.4%)		
6	SES	BPL (n=216)	20 (9.3%)	196 (90.7%)	0.386	0.765 (0.418-1.403)
		APL (n=238)	28 (11.8%)	210 (88.2%)		
7	Educational status of the mother	Low education (n=77)	9 (11.7%)	68 (88.3%)	0.727	1.147 (0.531-2.478)
		High education (n=377)	39 (10.3%)	338 (89.7%)		
8	Educational status of the father	Low education (n=113)	15 (13.3%)	98 (86.7%)	0.281	1.429 (0.745-2.74)
		High education (n=341)	33 (9.7%)	308 (90.3%)		
9	Occupational status of the mother	Low income (n=435)	47 (10.8%)	388 (89.2%)	0.442	2.180 (0.285-16.7)
		High income (n=19)	1 (5.3%)	18 (94.7%)		
10	Occupational status of the father	Low income (n=286)	34 (11.9%)	252 (88.1%)	0.234	1.484 (0.772-2.85)
		High income (n=168)	14 (8.3%)	154 (91.7%)		

Infection with one or more geohelminths was found in 48 of the 454 children studied, for an overall point prevalence of 9.69% (95% CI = 5.62-14.61). Specifically, the prevalence for A. lumbricoides, T. trichiura, and hookworms was 5.7%, 3.3%, and 0.9%, respectively. Aside from geohelminthic infection, 0.9% Enterobius vermicularis was found.

In sociodemographic variables, the bivariate analysis shows that children belonging to government schools and rural residences were favorably associated with geohelminthic infestation, which was statistically significant (p = 0.05). The odds ratio (95% CI) for government schools and rural residences was 2.136 (1.146-3.981) and 32.17 (4.3-235.5), respectively (Table 1).

There was no statistically significant relationship between geohelminthic infection and any variable related to the study participants' habits. However, wearing footwear while playing outside and attending personal hygiene classes in school were both protective against geohelminthic infection with a p-value of 0.20, so these two variables were used in the multivariate analysis.

Regarding the environmental variables, the source of drinking water was piped water, and the usage of their own latrine for defecation was statistically associated with geohelminthic infection, with p-values of 0.02 and 0.006, respectively. The risk factor was piped water, and the protective factor was using one's own latrine, with odds ratios of 1.95 (95% CI: 1.07-3.57) and 0.109 (0.026-0.453), respectively.

Table 2 describes the multivariate analysis between the risk factors and geohelminthic infestation. In the multivariate analysis, students living in rural areas (AOR = 40.16, 95% CI = 5.28-305.3), attending government schools (AOR = 3.06, 95% CI = 1.55-6.05), and drinking from piped water (AOR = 1.96, 95% CI = 1.01-3.77) were found to be significant risk factors for geohelminthic infestation. The model discovered that using the "own latrine" method of lavatory (AOR = 0.107, 95% CI = 0.022-0.522) and attending a personal hygiene class in school (AOR = 0.343, 95% CI = 0.142-0.832) were protective factors of geohelminthic infestation.

**Table 2:** Multivariate analysis to find the risk factors of geohelminthic infestations (Model Summary - Cox and Snell R2 for the model – 0.13.) *Statistically significant p-value

S. No	Variable	P value	Adjusted OR	95% CI
1	Rural residence	0.000*	40.16	5.28 – 305.3
2	Studied in a government school	0.001*	3.06	1.55 – 6.05
3	Always wearing Foot wear while playing outside	0.462	0.776	0.394 -1.52
4	Piped water for the source of drinking water	0.044*	1.96	1.01 – 3.77
5	Using the own latrine method of lavatory	0.006*	0.107	0.022 – 0.522
6	Had a personal hygiene class in school	0.018*	0.343	0.142 – 0.832

## Discussion

The overall prevalence of geohelminthic infection observed among schoolchildren (5-10 years) in the current study was 9.7%, with ascariasis at 5.7%, trichuriasis at 3.3%, and hookworm (Ancylostoma duodenale) at 0.7%. In addition to STH infection, 0.9% of Enterobius vermicularis was found. A study done by Farook MU et al. in 2002 reported a prevalence of 22.27% with 74.4% of ascariasis and 18.6% of hookworm infection in the tribal population of Thiruvananthapuram [7]. In Puducherry, a study was done among primary schoolchildren (5-10 years), which revealed ascariasis (43.21%), trichuriasis (10.9%), and hookworm infection (28.89%), respectively [9].

Compared to the evidence from the studies mentioned above, the prevalence of geohelminthic infestation among children in our study is much lower. In an Ethiopian study, it was hypothesized that the differences in prevalence among different communities might be associated with environmental sanitation, water supply, and the socio-economic status of the households [10]. This phenomenon of clustering can hamper attempts to control the infection, as individuals with heavy infections are likely to reintroduce it into the community. The reduction in prevalence could be attributed in part to a single-day mass drug administration campaign launched in specific districts of Kerala in 2005, during which diethylcarbamazine and albendazole were co-administered to all district residents on an annual basis. So far, seven rounds of MDA coverage in Kerala have been completed, including in the Thiruvananthapuram district. Increased public health awareness about the disease and improved personal hygiene practices were two of the reasons for the reduction in the prevalence of geohelminthic infections in Kerala.

Children belonging to government schools were associated with geohelminthic infestation with an odds ratio of 2.13 (95% CI: 1.14-3.98). Children residing in rural areas were associated with geohelminthic infestations with an odds ratio of 32.17 (95% CI: 4.3-235.5). Children who were studying in government schools and residing in rural areas function as a proxy measure of lower socioeconomic status, contributing to differences in geohelminthic infestation. This phenomenon is typical of developing countries. Anuar TS et al. found low-income households to be an important risk factor for soil-transmitted helminths, with an OR of 6.54 (1.53-27.9) [11]. In a study done in Ethiopia, urban residence was found to be a protective factor (OR: 0.45; 0.28-0.73) for geohelminthic infestation Though the proxy measures of low socioeconomic status were significantly associated with geohelminthic infestation, the socioeconomic classification based on above poverty line (APL) and BPL status did not show any significant association with geohelminthic infestation. The reason may be due to the misclassification of socioeconomic status based on many cumbersome criteria in Kerala.

According to our research, 97.6% of children wore footwear to school, but only 48.7% wore footwear while playing outside. Although footwear usage is reported to be a protective factor in many studies, there are some concerns that this could be an overestimate. In our study, questioning about hand washing habits and wearing footwear may lead to an overestimation of the practice, as the research participant might have the desire to provide a socially acceptable response and satisfy the interviewer. In the present study, no association between footwear usage and geohelminthic infection was noticed (OR: 0.77, 95% CI: 0.39-1.52, p: 0.462). A similar study in Vellore found that poor footwear usage did not significantly increase risk [12]. In contrast to a study conducted in the United States, a systematic review and meta-analysis discovered that children wearing shoes were protected against geohelminthic infection with an odds ratio of 0.30 (0.11-0.83) and that children who reported walking barefooted outside had higher infection intensities [13].

Children who attended personal hygiene classes in school were a protective factor for geohelminthic infection, with an adjusted odds ratio of 0.34 (0.14-0.83) reported in this study. In the Peruvian Amazon, a cluster randomized controlled trial study found that a school-based health hygiene education intervention was effective in increasing STH knowledge and reducing Ascaris lumbricoides infection [14]. Health education strategies have been found to reduce the cost of deworming and increase the level of overall health knowledge and acceptability of deworming interventions within the community. So the benefits of school-based periodic deworming programs are likely to be enhanced when a sustained health hygiene education intervention is integrated into the school curriculum.

This study found that households that used piped water from the corporation had a lower risk of geohelminthic infection. The adjusted odds ratio for using piped water was 1.96 (95% CI: 1.01-3.77) compared to those using their own well water for drinking purposes. A similar finding was also observed in a study done in Ethiopia, which found that using water from a pipe inside a compound was a risk factor for helminthic infection [15]. This phenomenon is typical of low- and middle-income countries. But a systematic review and meta-analysis study done in the US found piped water access did not lower the odds of geohelminthic infection with an OR of 0.93 (0.28-3.11), but the same study found piped water did lower the risk of individual A. lumbricoides and T. trichiura but not any STH infection [13]. This might be due to the poor quality of piped water and the high chance of contamination. Efforts to minimize microbial contamination of piped water supplies and to monitor water quality are important for controlling geohelminthic infection. However, other unknown factors may contribute to the increased risk associated with piped water and merit further investigation.

The use of a private latrine for defecation was found to be a protective factor for geohelminthic infestation when compared to those who used a public toilet or open-air defecation, with an adjusted odds ratio of 0.107 (95% CI: 0.02-0.52). A strong association between toilet usage and decreased STH infections has been demonstrated in several studies in developing countries. A systematic review of 36 studies revealed toilet usage had a significant overall protective effect of about 51% for STH infections in general and 60% for hookworm infections [13].

Limitations and strength

The limitation of this study is the single stool examination for the detection of intestinal parasites, which could have underestimated the prevalence, and we could not draw conclusions on the causality of associations of different factors with geohelminthic infection, as this is a limitation of the cross-sectional study design. The fathers or guardians who answered the children's habit-related questions may not have given the exact, correct answer, so the subjective variation of answers was a limitation of the study. But this study is the first, to our knowledge, to explore the prevalence of and risk factors for geohelminthic infection in Kerala, and focusing on this age category (5 to 10 years) is its strength.

## Conclusions

The prevalence of geohelminthic infestation among schoolchildren in this study was 9.7%. Individual species prevalence of A. lumbricoides, T. trichiura, and hookworm were 5.7%, 3.3%, and 0.7%, respectively. In addition to geohelminthic infestation, this study discovered a prevalence of 0.9% with Enterobius vermicularis. The factors found to be significantly associated with geohelminthic infestations after doing a multivariate analysis were children belonging to government schools, residing in rural areas, and using piped water as a source of drinking water. Those who were using their own latrines for defecation and students who attended personal hygiene classes in school were found to be protective factors for geohelminthic infestation.
